# The Initial Assessment of Daily Insulin Dose in Chinese Newly Diagnosed Type 2 Diabetes

**DOI:** 10.1155/2016/7245947

**Published:** 2015-12-01

**Authors:** Jing Ma, Huan Zhou, Hua Xu, Xie Chen, Xiangyu Teng, Qianjing Liu, Wei Liu

**Affiliations:** Department of Endocrinology and Metabolism, Renji Hospital, School of Medicine, Shanghai Jiao Tong University, No. 1630 Dongfang Road, Shanghai 200127, China

## Abstract

*Background*. It has been well accepted that insulin therapy is the ideal treatment for newly diagnosed diabetic patients. However, there was no study about assessment of the initial insulin dosage in new onset Chinese patients with type 2 diabetes. *Research Design and Methods*. 65 newly diagnosed patients with type 2 diabetes (39 males/26 females; HbA1c ≥ 11.80 ± 0.22%) were investigated. All patients had random hyperglycaemia (at 21.8 ± 3.9 mmol/L) on the first day of admission and received insulin infusion intravenously (5 U/per hour). When the blood glucose level dropped to around 10 mmol/L, patients were then transferred to continuous subcutaneous insulin infusion (CSII). The reduction of blood glucose levels in response to per unit of insulin (RBG/RI) was recorded. The target glucose level was achieved in about 3 days. The total daily insulin dose (TDD) and basal insulin dose (TBD) were calculated. *Results*. TDD was 45.97 ± 1.28 units and TBD was 19.00 ± 0.54 units. TBD was about 40% of the total daily insulin requirement. There was a negative correlation between the ratio of RBG/RI and TDD. *Conclusions*. TDD was correlated with blood glucose reduction in response to intravenous insulin infusion in Chinese new onset patients with type 2 diabetes.

## 1. Introduction

It has been reported that the prevalence of diabetes in Chinese adults was up to 11.6% by the China Noncommunicable Disease Surveillance Group in 2013 [1]. Patients with type 2 diabetes in Asian country mainly have impaired *β*-cell function rather than insulin resistance compared to those in western country [2]. Therefore, early intensive insulin therapy is used as the ideal therapy to restore the *β*-cell function and to eliminate glucotoxicity in patients with newly diagnosed type 2 diabetes [3, 4].

Intensive insulin therapy normally includes continuous subcutaneous insulin infusion (CSII) and multi-daily insulin injections therapy (MDI). Patients with CSII present more prominent reduction in HbA1c and glycaemia with less hypoglycaemic events compared to MDI [5]. CSII is widely used in patients with type 1 diabetes, pregnant women with gestational diabetes mellitus, and patients with type 2 diabetes in preoperation or those with poor glycaemic control [6]. Optimal insulin pump therapy is determined by accurate setting of total daily insulin dose (TDD), basal and bolus insulin dose [7]. It is primary to get the TDD which is normally estimated by body weight (0.4–0.5 IU/kg per day) according to the Chinese guideline of insulin pump in 2010 [8]. Basal insulin dose (TBD) is calculated by the certain ratio of TDD. It has been shown in a Japanese study that TBD is about 30% of TDD in patients with type 1 diabetes who use the insulin pump [9]. The bolus dosage is adjusted according to carbohydrate factor (CarbF) with “450 rule” and glucose correction factor (CorrF) with “1500 rule.” However, there was no study about the initial insulin dose in newly diagnosed patients with type 2 diabetes.

The purpose of this study was to assess total daily insulin requirement and the impact factors in newly diagnosed patients with type 2 diabetes, who were on transient CSII therapy in China.

## 2. Methods

### 2.1. Subjects (Table 1)

All participants with type 2 diabetes were diagnosed by World Health Organization criteria. None had eating disorders, diabetic ketoacidosis, hyperosmolar status, and other acute diabetic complications. All patients have the negative GAD antibody levels.The anion gap in the initial electrolytes was 12.2 ± 1.6. (The normal range is 8–16.) They have the characteristics as follows: 39 males/26 females; age 49.72 ± 2.1 years old, body weight 66.5 ± 1.6 kg, height 165.6 ± 1.1 cm, BMI 24.2 ± 0.4 kg/m², and glycated haemoglobin (HbA1c) 11.80 ± 0.2%.

### 2.2. Protocol

A total of 65 Chinese newly diagnosed patients with type 2 diabetes were included in this study. All the patients were hospitalized in Department of Endocrinology and Metabolism of Shanghai Renji Hospital between July 2011 and December 2013. All the patients had random postprandial glucose levels at 21.8 ± 3.9 mmol/L on the first day of admission. They were given intravenous insulin infusion at the initial rate of 5 U/h (25 U insulin/250 mL saline). When patients received intravenous insulin infusion, none of antidiabetic mediations were used. We monitored the capillary blood glucose every 30 minutes. The intravenous insulin infusion was stopped when the capillary glucose levels were dropped to around 10 mmol/L. The reduction of blood glucose and the dose of insulin were recorded and the ratio of the reduction of blood glucose/per unit of insulin (RBG/RI) was calculated. All patients were switched to insulin pump (Paradigm 712 pump, Medtronic, Northridge, CA) with rapid-acting insulin (Insulin Aspart Injection, Novo Nordisk) for 72 hours. Capillary blood glucose levels were tested eight times daily (before meal, after meal, bedtime, and 2 a.m.). In 72 hours, the basal insulin rate and the bolus insulin dose were adjusted according to the capillary blood glucose levels. The target fasting glucose and 2 h postprandial glucose were set at 7 mmol/L and 10 mmol/L. At the end of 72 hours, all the patients got the target glycaemia. The insulin pump was stopped. TBD and bolus insulin dose for all of them were recorded. During the hospitalization, the meals were prepared by dietitians (total energy intake 20–25 kcal/kg, 50–60% from carbohydrate, 15–20% from protein, and 20–25% from fat). No additional food was consumed unless hypoglycaemic events occurred.

### 2.3. Statistical Analysis

The data were shown as means ± standard error. The mainly statistical methods included bivariate correlation analysis and multiple regression analysis with SPSS 20.0. *P* values < 0.05 were considered significant.

## 3. Results

### 3.1. Insulin Dose

When the target glucose level was achieved, the TDD was 45.97 ± 1.28 U and TBD was 19.00 ± 0.54 U. The percentage of TBD was 41.74 ± 0.87%. Total bolus dose was 27.01 ± 0.96 U (prebreakfast bolus 11.87 ± 0.44 U, prelunch bolus 6.79 ± 0.29 U, and predinner bolus 8.35 ± 0.36 U). The percentage of prebreakfast, prelunch, and predinner bolus to total bolus dose was 44.15 ± 0.71%, 25.19 ± 0.58%, and 30.65 ± 0.65%, respectively.

### 3.2. Correlation between the Ratio of RBG/RI and Insulin Dose

**Figure 1:**
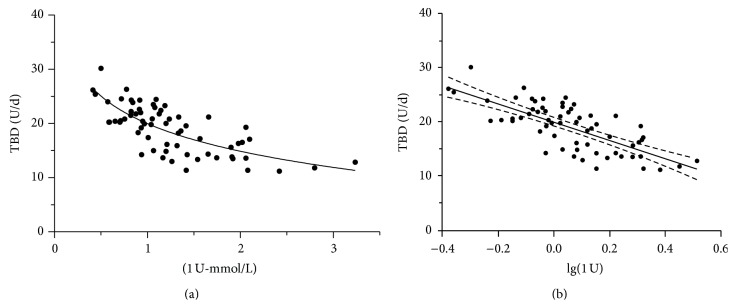
(a) TBD was decreased with the ratio of RBG/RII: *r* = −0.710 (*n* = 65), *P* < 0.01. (b) Correlations of the ratio of RBG/RII (log transformed) with TBD in 65 subjects: *r* = 0.740, *P* < 0.01.

There was a negative correlation between the ratio of RBG/RI and TBD (*r* = −0.710, *P* < 0.01) and TDD (*r* = −0.546, *P* < 0.01). However, there was no relationship between the ratio of RBG/RI and total bolus dose, but the ratio of RBG/RI was negatively related to prebreakfast (−0.320, *P* < 0.01) and prelunch bolus (−0.292, *P* < 0.05). It tended to be related to predinner bolus (*r* = −0.211). TBD presented logarithm relevant to the ratio of RBG/RII (*r* = −0.740, *P* < 0.01) (Figure 1). Body weight, BMI, fasting blood glucose, and TG were related to the TDD and TBD. However, HbA1c was not the independent factors for TDD or TBD.

### 3.3. Stepwise Multiple Regression Analysis

TBD was independently correlated with lg (the ratio of RBG/RII) (*β* = −0.675, *P* < 0.01) and age (*β* = −0.377, *P* < 0.05) with stepwise multiple regression analysis. Regression equation was *y*(TBD) = 25.62 − 15.927 *∗* lg[x1 (the ratio of RBG/RII)] − 0.101 *∗* x2 (age).

## 4. Discussion

Our study is the first to show that the initial dose of insulin is related to the ratio of RBG/RI in Chinese new onset patients with diabetes who use the insulin pump therapy. The basal insulin dose is about 40% of total daily insulin requirement.

In newly diagnosed Chinese patients with type 2 diabetes, insulin secretion, in particular at early phase, is normally reduced due to the impaired *β*-cell function [10]. Hyperglycaemia caused glucotoxicity which leaded to further decline of *β*-cell function [11]. Patients with type 2 diabetes enrolled in our study were drug-naïve with random hyperglycaemia (blood glucose levels 21.8 ± 3.9 mmol/L and HbA1c 11.8 ± 0.2% as shown in Table 1). BMI was in the range of 23.8 to 24.2 kg/m² (as shown in Table 1). According to the Chinese Adult Obesity Guide, overweight is defined as BMI over 24 kg/m^2^. Therefore, patients in our study were normal body weight or slightly overweight. It appears primary for those diabetic patients to obtain the target glucose level and reserve *β*-cell function with insulin therapy.

Intravenous infusion of insulin was previously used to achieve glycaemic target for patients in the Intensive Care Unit [12] and those with diabetic ketoacidosis due to the rapid action and shorter half-life [13]. In order to lower blood glucose level in the shorter period, we infused insulin intravenously before insulin pump therapy. Although the decline rate of glucose levels may be related to many factors such as the relative degree of insulin resistance, the differences in the clearance of glucose in the urine, and the amount of fluid infused or ingested into the patient, the RBG/RI ratio has the strongest correlation with TBD among all those factors (Figure 1). We aimed to find a practical method in calculation of the initial dose of insulin in the clinical work. Blood glucose levels at admission were fairly high. Insulin sensitive factor itself would be variable due to glucotoxicity. It was not reasonable to explore the relationship of *β*-cell function with TDD during the 72 hours of CSII. It is not surprising that body weight was correlated with TDD. We estimated the insulin dose according to the body weight (0.4–0.5 IU/kg per day) in previous calculation. We also found that TBD was independently correlated with lg (the ratio of RBG/RII) and age with stepwise multiple regression analysis (as shown in Figure 1). Therefore, we did regression equation as *y*(TBD = 25.62 − 15.927 *∗* lg(the ratio of RBG/RI) − 0.101 *∗* age) by the software. It may provide the direction that we could easily calculate the insulin dose in the future clinical work.

Chinese has changed eating habit from high-carbohydrate to high protein/fat dietary pattern in recent years. However, Chinese populations still have rice and noodle as major energy resource, particularly in breakfast. It has been reported that newly diagnosed Chinese patients with type 2 diabetes consumed higher proportion of carbohydrates (over 65%) in the diet compared to that recommended by Chinese and international dietary guidelines for macronutrients [14]. It is not surprising that Chinese new onset diabetic patients have higher occurrence of the isolated postprandial hyperglycaemia than fasting hyperglycaemia (44.1% of the men and 50.2% of the women) [15]. Accordingly, the percentage of bolus insulin dose was higher in Chinese population than that in western country.

This study has some limitations. Firstly, we did not use a continuous glucose monitoring system (CGMS) before and after the treatment in our study. However, it has been shown that eight-point glucose test daily is comparable to CGMS [16]. Secondly, we did not clarify the correlation of energy intake with premeal bolus of insulin. The dietitian prepared the meal according to the body weight. We already found that there was a weak correlation between body weight and insulin dose. Finally, the duration of this study was relatively short. Longer term studies and larger studies are required in the future research.

In conclusion, basal insulin requirement is 40% TDD in newly diagnosed patients with type 2 diabetes in China. TDD was related to the rate of blood glucose reduction in response to initial intravenous insulin infusion.

## Figures and Tables

**Table 1:** Clinical characteristics of the subjects.

Variables	Value
M/F (*n*)	39/26
Age (years)	49.7 ± 2.1
Weight (kg)	66.52 ± 1.56
Height (cm)	165.6 ± 1.08
BMI (kg/m²)	24.15 ± 0.43
WHR	0.96 (0.89–0.96)
HbA1c (%)	11.8 ± 0.22
FCP (ng/mL)	1.53 (0.96–2.52)
Cr (*μ*mol/L)	58.49 (41.80–68.95)
BUN (mmol/L)	6.54 ± 0.72
UA (*μ*mol/L)	296.4 ± 14.65
TC (mmol/L)	5.48 ± 0.22
TG (mmol/L)	2.08 (1.09–2.69)
LDL (mmol/L)	3.54 ± 0.20

Data represent means ± SE, or median (interquartile range 25–75%).
